# Intervening to prevent suicide at railway locations: findings from a qualitative study with front-line staff and rail commuters

**DOI:** 10.1192/bjo.2022.27

**Published:** 2022-03-09

**Authors:** Dafni Katsampa, Jay-Marie Mackenzie, Ioana Crivatu, Lisa Marzano

**Affiliations:** Department of Psychology, Middlesex University, UK; Department of Psychology, Westminster University, UK; Department of Psychology, University of Suffolk, UK; Department of Psychology, Middlesex University, UK

**Keywords:** Suicide, qualitative research, bystander interventions, railways, public interventions

## Abstract

**Background:**

For every suicide on the British railway network, at least six potential attempts are interrupted by front-line staff or rail commuters. However, the factors that maximise or hinder the likelihood and effectiveness of such interventions are poorly understood.

**Aims:**

The aim of the current study was to shed light on the experience of intervening to prevent a suicide at a railway location, including how and why people intervene, and their feelings and reflections in the aftermath.

**Method:**

In-depth interviews were carried out with rail commuters (*n* = 11) and front-line railway staff (*n* = 10) who had intervened to stop a suicide by train. Data were analysed thematically.

**Results:**

Participants had intervened to prevent suicide in several ways, both from afar (e.g. by calling a member of staff) and more directly (verbally or non-verbally), in some cases with no prior training or experience in suicide prevention, and often as a ‘quick, gut reaction’ given the limited time to intervene. In more ‘reasoned’ interventions, poor confidence and concerns around safety were the greatest barriers to action. Although often privy to their final outcome, most participants reflected positively on their intervention/s, stressing the importance of training and teamwork, as well as small talk and non-judgemental listening.

**Conclusions:**

Suicides in railway environments can present bystanders with little time to intervene. Potential interveners should therefore be resourced as best as possible through clear infrastructure help/emergency points, visibility of station staff and training for gatekeepers.

## Background

Every year around 6000 suicides are recorded in the UK, and during 2018–2019, there was a significant increase (11.8%) in the rate of suicides for the first time since 2013.^1^ Around one in 20 of these deaths occurred on the British railway network,^2^ with profound implications for all those affected by these tragic events, including bereaved family and friends, train drivers and other witnesses. Recent evidence suggests that this proportion could be substantially higher but for the circa 1800 ‘life-saving’ interventions recorded yearly on the railways in the UK.^2^ Thus, for every life lost by this method, at least six appear to be saved by a third-party intervention involving railway staff, emergency services and/or members of the public. However, the factors (or combination of factors) that maximise or hinder the likelihood and effectiveness of such interventions are poorly understood.

Research has begun to explore these questions in relation to high-frequency suicide locations and so-called ‘hotspots’, as well as public locations more generally, although a 2015 systematic review identified no studies assessing measures to increase third-party interventions as a standalone strategy at suicide hotspots.^3^ More recently, Owens et al^4^ conducted a qualitative analysis of interventions by ‘passing strangers’ and concluded that lay people should be encouraged to reach out to those in crisis in public places, as this required no special skills or saying the ‘right thing’, and could be done effectively even when spontaneous and unscripted. Nonetheless, the authors also noted that different social contexts and stages of the suicidal process may require different approaches, and that interveners can be left feeling taxed, troubled or worse (see also Mitchell et al^5^). Exploring their experiences before, during and after an intervention is thus an important aspect of understanding how interventions to prevent suicide may be made safely, for all concerned.

Compared with many public locations, railway environments are arguably unique. A separate, focused analysis of life-saving interventions in this context is therefore warranted. For example, the close proximity to lethal means of suicide (and potential for injury or even death for a person making an intervention) in railway locations compared with many other public places may influence a person's decision to intervene, by reducing the ambiguity of emergency situations^6,7^ as well as bystanders’ perceived safety and behavioural control.^8^ In addition, crowd behaviours and interactions (or lack of) in railway settings are often anonymous and transient by their very nature.^9^ Bystander anonymity and lack of group cohesiveness are known inhibitors of helping behaviours in a variety of emergency and non-emergency contexts.^6,10^ Also, the presence (or possibility of presence) of staff may contribute to the perception of rail locations as being less ‘public’ than spaces such as unstaffed bridges or costal cliffs. This may, in turn, discourage members of the public from intervening when a fellow commuter appears to be in danger,^11^ but it also offers the potential to ‘delegate’^12^ and train an important group of likely ‘gatekeepers’ (e.g. Marzano et al^13^). Indeed, both in Australia^7^ and the UK,^14^ approximately 90% of life-saving interventions in railway settings are made by rail personnel or emergency services as opposed to members of the public. Clearly, it is important to explore the experiences and perspectives of all of these groups, to fully understand where, how and when suicide attempts at rail locations are more likely to be safely and effectively interrupted by a third party.

## Current literature

To date, the limited but growing literature on bystander interventions in the context of suicidal behaviour has tended to concentrate on interventions with family and close peers.^5,15,16^ Only one study has specifically examined the role of bystanders in the prevention of rail suicide attempts, but its primary focus was on interventions by members of the public, based on a descriptive analysis of official records in New South Wales, Australia.^7^ Along with the wider literature on helping behaviours in dangerous public situations,^10^ these studies suggest that bystanders are more likely to intervene to help than is commonly feared, particularly when the ambiguity of danger is low. Thus, ‘it is time to change the narrative away from an absence of help and toward a new understanding of what makes intervention successful or unsuccessful’^15^ from the perspective of those making, as well as receiving, such interventions. As part of a wider study of interventions to prevent suicides at railway locations, we therefore carried out in-depth interviews with staff and rail commuters who reported having intervened to stop a suicide by train. With so little currently known about their experiences, and potentially so much to be learnt from them to prevent more suicides at rail locations, our key aims were to understand how participants intervened, what contributed to their decision to intervene and how they felt after their intervention.

## Method

### Participants

Through a national cross-sectional survey, we identified UK-based men and women over 16 years of age, who reported having ‘intervened is some way in a situation when someone around you appeared to be suicidal at a train/tube station or tracks, for example by talking to them, asking if they are ok, calling for help or attempting to interrupt or make contact with them in some other way’, and had expressed an interest in participating in a follow-up interview about their experiences of this. Written informed consent was obtained from all participants before the interviews.

As our key aim was to capture the stories of a range of groups and individuals involved in suicide interventions on the railways, we advertised the survey to rail commuters and staff, as well as the wider general public, both online (particularly, but not exclusively, within special interest groups and networks with a focus on suicide prevention, mental health and UK railways) and via posters and leaflets on university campuses and at busy railway locations. The survey, and then interview, sample therefore included both members of the public and rail employees (with no exclusion criteria relating to personal or professional backgrounds, or lived experiences of suicidality, although we discouraged participation from those experiencing strong thoughts of suicide or who had attempted suicide in the past month).

### Materials and procedures

In consultation with Samaritans, rail industry stakeholders and the project advisory group (which included people with lived experience of suicidality), we developed a semi-structured interview schedule to explore people's thoughts, feelings and experiences before, during and after intervening at a railway location (see COREQ in the Supplementary Material for further information). During the interviews, participants were encouraged to give free-narrative responses, with prompting and follow-up questions where this was felt to be appropriate. Interviews were conducted face to face at Middlesex University or local Samaritans branches (*n* = 8), over the telephone (*n* = 9) or via Skype (*n* = 4), between April and November 2019.

### Ethics

The authors assert that all procedures contributing to this work comply with the ethical standards of the relevant national and institutional committees on human experimentation and with the Helsinki Declaration of 1975, as revised in 2008. All research materials and procedures were approved by the Psychology Department Research Ethics Committee at Middlesex University (ethical approval reference: 2019 Feb.7045).

### Analysis

Interviews were audio-recorded, transcribed verbatim and anonymised, before conducting an inductive thematic analysis following the six stages recommended by Braun and Clarke:^17^ becoming familiar with the data, generating initial codes, exploring both sematic and latent themes within the data, reviewing themes, defining and naming themes, and write-up. Final identification of themes was based on consensus discussions between members of the research team. Main themes and subthemes were identified across the whole sample and then specifically in relation to rail commuters and front-line staff (including railway employees and police), to highlight meaningful differences and similarities. In the following section, direct quotations from participants’ interviews are included to illustrate key themes and subthemes (with ‘C’ suffixes denoting commuters and ‘S’ denoting front-line rail and emergency staff).

## Results

The final interview sample included 16 males and five females who had intervened when someone around them appeared to be at risk of suicide at a railway location. This included 11 members of the public (of whom four also had lived experience of suicidality and three were mental health professionals) and ten front-line staff (including six train drivers, three railway employees and one police negotiator). All participants described themselves as White British, with ages ranging from 23 to 62 years (mean age 48 years).

The key aim of the interviews was to explore individual experiences of carrying out an intervention, and therefore, what our participants defined as an intervention, why they made the decision to intervene and the feelings they experienced in relation to the intervention(s) they had made. Main themes and subthemes in relation to these are presented in Table 1 below.

**Table 1:** Main interview themes and subthemes

Main themes	Subthemes
What is an intervention?	Up-close and interactive interventions Interventions from afar The value of teamwork
Deciding to intervene: gut instinct versus calculated decision	Quick: ‘an instantaneous decision’ Considered interventions
Looking back: feelings about intervening	Hindsight Interventions without endings

### Theme 1: what is an intervention at a railway location?

In participants’ accounts, an intervention took different forms; sometimes interventions were up-close and personal, but other times interventions involved practical tasks carried out away from the suicidal person. Participants expressed that an intervention was not always something that involved one person or one intervener; teamwork was often deemed to be essential.

#### Up-close and interactive interventions

Participants emphasised the importance of talking to the individual in a calm manner, and helping them feel listened to. Small talk, active listening, asking questions and trust building were felt to be useful intervention techniques:

‘It's just being able to listen to people, and trying to understand … you'll never understand why they're in the place they're in because there's professionals that do that, but it's just getting them down to that … so yeah, it's just being able to remain calm … ’ (S4).

In some instances, any distraction was viewed as being potentially helpful:

‘I stopped and spoke, pure and simply just to try and distract her really from doing what she was potentially going to do’ (S6).

Distractions also included non-verbal interactions such as eye contact, smiling or standing close to someone. These were viewed as particularly useful types of intervention if the person did not appear to be in immediate danger, and were sometimes used as a way to instigate contact:

‘The non-verbal's really interesting because I know I've certainly done it before with incidents where we've turned up and we haven't said anything and we've just stood there, quite … fairly close to an individual and allowed them to start the conversation, just smiled, just looked at them and smiled, made eye contact’ (S21).

However, participants felt that visually monitoring the person's behaviour was also needed. During some interventions participants recalled using their body as an obstacle to prevent the person getting near to or on the track:

‘ … by standing in front of her, so she would have had to have gone round me [ … ] So I could force her into a position where she couldn't actually get direct access. If you've got that spatial awareness with you at the time, that's really handy’ (C3).

Physical interventions such as restraining someone were viewed as a last resort and something to be avoided if possible: ‘and probably I would say no physical contact to start with … ’ (S8). Professionals recognised the need for physical restraint more than commuters, but were also aware that an intervention should be managed emphatically, and that there are situations when physical interventions may make the situation worse:

‘So it's … it's just physical. And then you're in that sort of, that issue of how, you know, are you then the assaulter rather than the intervener? Mm. So it's things like that, you know, I'm not saying it shouldn't be done but it should be the last thing that's done’ (C3).

#### Interventions from afar

Some interventions involved limited or no contact with the suicidal person. For example, interventions by train drivers included practical means of informing colleagues at the upcoming station, calling in an emergency and bringing the train to an immediate stand: ‘We have a procedure, we make an emergency call, stop the lines, the police are notified’ (S6).

Calling for professional help (e.g. railway staff, British Transport Police or other emergency services), rather than directly approaching the person, was considered a crucial form of intervention:

‘I think the best thing to do … would be to seek support, like a railway staff support of police support or something like that’ (C7).

Some participants mentioned the importance of infrastructure points that may help encourage members of the public to seek professional assistance when concerned about someone (e.g. emergency phones), especially if they lack the confidence to engage directly with the person in distress:

‘I think you need the structures in place, like you know not everyone is going to be able to physically stop someone or drag someone off, like so … for heaven's sake, have lots of emergency buttons that are accessible! [ … ] that you press and the train driver knows there's something happening’ (C10).

#### Value of teamwork

An intervention often involved not just one person, but multiple people. Participants felt this made the intervention more manageable:

‘The guard was helping [ … ] another person to speak to him and try and calm him down, and then the nurse helped because he actually took over from what I was doing and allowed me to make a couple of calls which related actually to the operation of the train, which was obviously my responsibility at the time. I felt we had a very good example of team work’(S20).

‘You're not going to be a bystander and solve it … On your own, yeah. … on your own, you are going to have to communicate with staff. So it's telling people … And I guess I think staff because whether that's staff or police or … But if you do speak to someone and they are suicidal, surely at some point you're going to need to get professionals involved’ (C5).

Although actively seeking support from railway staff was a recurrent theme among commuter participants (‘ … and then someone from British Transport Police came and then they were speaking to him, so I went and got my train to leave’ (C2)), the important contribution made by lay bystanders was also stressed:

‘You've automatically got that barrier haven't you with the police uniform on. I think people think if they see the police they're going to get taken away or ehm … locked up in hospital and … But if were just Joe Public going about your daily business and have a chat to somebody that you feel might be in some sort of position of danger, I think it's a lot … I'm only speculating, I feel it would be a lot more effective than ehm … having someone in uniform telling you … ’ (S17).

### Theme 2: deciding to intervene: gut instinct versus calculated decision

Participants expressed several driving forces behind why they chose to intervene, which was broadly dictated by the time they had to process the situation. This included a quick ‘gut reaction’ intervention in which they had little to no time to make an informed decision about whether or not to intervene versus a more time-generous, calculated intervention.

#### Quick: ‘an instantaneous decision’

Participants expressed that some situations appeared urgent and required immediate action: ‘In a split second, in an instantaneous decision, I made a decision’ (S20). In these scenarios, participants expressed having a ‘natural, gut reaction’ (C7):

‘I think normal human instinct, gut instinct, you can quickly tell things aren't going to be right or something's not right about the whole scenario’ (S4).

#### Considered interventions

Some interventions afforded participants more time to consider whether to intervene. In bystanders’ accounts, the first factor influencing this process was how safe an intervention may be for themselves and the person in distress:

‘You make a decision whether or not to speak to them, and that's partly your own health and safety’ (C10).

‘[ … ] the likelihood of him falling or jumping off this [rail] bridge is high, and the likelihood of death is high. The likelihood of him fighting me back is moderate. The likelihood of me being injured if he fights me back is moderate. So it's looking at the severity of outcome, multiplied by the likelihood of it happening, the risk was, would have been greater not to intervene’ (C2).

A further factor was the participants’ confidence in approaching the person, particularly for members of the public:

‘Let's say I intervene and the [individual] tells me to bugger off and leave me alone, and then it's a bit embarrassing for me…looking like an interfering fool! So there's a bit of potential for public shaming’ (C1).

Even if participants were certain about the person being in distress, they also expressed a sense of nervousness around having the confidence and skills to communicate effectively with them. Having made a previous intervention, or received training in relation to mental health, were seen to increase such confidence:

‘You develop a sort of radar for the unusual or the people that when something just doesn't sit quite right’ (S8).

‘Having the confidence will only come by having the awareness and the adequate training to actually do it’ (S6).

‘I mean I had confidence in my ability because of my training and things that you know should I get down there. And I can, you know I can stop the trains’ (S9).

Participants also expressed a feeling of responsibility toward the person in distress, which appeared to be amplified for those with professional experience:

‘I was confident actually that it was my professional responsibility to intervene, as well as my moral responsibility’ (S20).

‘We've got there and because they know we're all Samaritans trained, have the managing suicidal contact [training], they kind of stood back and let us took over’ (S11).

### Theme 3: looking back

The majority of participants reflected positively on their intervention/s, expressing that it was the right course of action and responsible thing to do:

‘Whether or not I helped or not I have no way of knowing, but my intention was positive and I acted on it, so I feel in principle good about that, that I took some action, with good intentions’ (C1).

‘I was quite happy with my response … my reactions to the situation because if I didn't … But yeah, I was overall pleased with my reactions and how I communicated to the signaller … ’ (S4).

In some cases, the intervention was also considered to be a stepping stone toward the person getting professional support:

‘So I'm hoping that you know just by stopping and talking to her, gave the authorities chance to get there and talk her out of it’ (S6).

However, some participants questioned whether an alternative route of action could have been taken, or if they should have behaved differently:

‘I would have looked and sounded a little more natural and at ease [ … ] If I had a bit more courage in my convictions earlier, I might have intervened [earlier]’ (C1).

‘I would actually approach railway staff first of all in those circumstances and say, look, there's someone sitting on the end there, a bit concerned about their welfare. And approach them with the railway staff, I wouldn't try and do that all on my own [again]’ (C7).

#### Interventions without endings

Several interviewees described their intervention as an ‘unfinished story’, and at times struggled with not knowing what happened after they intervened:

‘I'd like to have stayed a bit later to see like you know the outcome of it and make sure the police did you know talk her down, but I was limited, I had to go to work’ (S6).

‘I did try and check the news later to see if there'd been any fatalities on the network and I couldn't see anything’ (C1).

‘The thoughts I had afterwards, they were sort of fantasies about what happened to him. Was he alright?’ (C2).

For some participants, the intervention/s made had had a lasting impact, particularly if they had also experienced a fatality on the railways: ‘there is always the fear that they're going to come back and try it again’ (S19). A death by suicide on the railway can be a traumatic event for train drivers and other witnesses: ‘I think about it every day still, to this day … I was off work for five months because it took me a long time to accept’ (S4). For some, avoiding such trauma had then become the main motivation for intervening to prevent further suicides:

‘It's not just the drivers and the train crew, it's the emergency services and you know the families, it's just I don't want anyone having to go through it. [ … ] I think that's what other people don't seem to realise, the impact it has on everyone else’ (S4).

## Discussion

Third-party interventions to prevent suicide are a daily but under-researched occurrence at railway locations across the UK. In an effort to capture people's experiences of conducting a potentially life-saving intervention in this context, we interviewed ten front-line staff and 11 commuters who had intervened to prevent a suicide by train. Our findings suggest that such interventions are often complex and multifaceted events, which can take several forms and involve a range of people, including bystanders, trained professionals and rail employees. These themes were echoed in the accounts of both professional and lay interveners, although the former appeared to place more value on the use of physical restraint as a last resort. Regardless of the nature of the intervention itself (e.g. whether or how much direct contact with the person in distress was reported), the value of teamwork and the importance of summoning help from others, especially staff, emerged as key themes throughout the interviews. The latter was a particularly recurrent theme among commuter participants, whereas professional interviewees appeared to have an increased sense of their own responsibility to intervene. In other words, railway staff and first responders were seen – and appeared to see themselves – as having a central, ‘gatekeeper’ role in suicide prevention and interventions. This marks a potentially important distinction between third-party interventions on the railways versus more ‘public’, unstaffed locations; and a potential challenge at railway locations where rail employees are not always present, visible or easy to reach.

Nonetheless, our findings also support earlier research in suggesting that lay people can and do play an important part in suicide prevention,^4^ and that this does not necessarily require direct contact, be it verbal or physical, with people in distress. Indeed, their very presence (even in the absence of an ‘intervention’, as such) may act as a potential deterrent for those considering taking their own lives,^18,19^ and, at least for some, a less threatening presence than uniformed staff (especially police). Some interviewees discussed intervening ‘from afar’ or non-verbally (for example, by using their body language or positioning, or simply calling a member of staff). This is arguably an important message for public awareness campaigns aiming to increase the likelihood of bystanders intervening to prevent suicide, particularly for those lacking the confidence to initiate contact with a potentially suicidal individual.

As in previous studies,^4,20,21–23^ lack of confidence was the greatest barrier to intervention identified by our sample, and training was often described as the best anecdote against it.^24–27^ Being trained in ‘managing suicidal contacts’ was said to affect preparedness to act when an individual is distressed, and the ability to identify suicide risk in the first place, although this was also described as a natural human instinct. This clearly supports gatekeeper training initiatives for front-line staff at railway locations^13^ and more widely,^28,29^ and the importance of evidence-based guidance on suicide intervention being easily and freely available and accessible;^16^ if only to reassure the general public that the first steps in helping someone who might be suicidal need not require specialist skills in suicide prevention, or indeed direct questions about suicide.^4^

Being able to quickly assess and manage the wider risks that may be associated with an intervention on the railways (not least from a health and safety perspective) was also described as key. Although some of the knowledge and actions associated with such risk assessment would arguably fall outside what a member of the public would be expected to know or do (e.g. to stop a train), having clear and visible infrastructure points at key locations, and sufficient guidance on how these should be used in an emergency (not only or necessarily relating to suicide), could make a real difference to lay bystanders’ ability to summon expert help when required (provided, of course, that this is available and resourced for a prompt and efficient response).

Further underscoring the importance of gatekeeper training and the need for clear processes and infrastructure to raise alarm in an emergency (including at unstaffed and remote locations) is the often small window for intervention available. The intervention experiences described by interviewees were not always planned or carefully considered, as there was very little time to evaluate whether or how to intervene. Prior knowledge of what to do in a situation like this is therefore essential, as is the availability of support in its aftermath, where needed.

Our findings support earlier literature in suggesting that intervening to prevent suicide can feel unsettling, unresolved or even traumatic,^4^ especially (but not exclusively) when the outcome of the intervention is negative or unknown. Indeed, although mostly positive about their attempts to prevent suicide, many participants described this as a challengingly ‘unfinished story’. Of course, it may not be feasible, appropriate or even legal for interveners to be informed of how the story actually unfolded, but a space to reflect on what happened (and what might have happened) could be beneficial. This could be embedded within a reflective practice framework for front-line staff,^30^ whereas ‘lay’ interveners could, at a minimum, be directed to other organisations (e.g. Samaritans) that support people dealing with difficult experiences and emotions. Trauma debriefing and more structured psychological support may be required in some cases (e.g. following an unsuccessful intervention), but current evidence points against their use in the immediate aftermath of trauma.^31^

### Strengths and limitations

This is the first study to focus on the perspectives of lay bystanders, mental health professionals and front-line staff who have intervened to prevent suicide at railway locations. Using a qualitative methodology enabled a rich and nuanced understanding of intervention experiences in this context, but the relative lack of diversity in our sample and its self-selected nature are clear limitations, along with issues of poor recall and self-presentation biases. Our findings may not necessarily be generalisable to all suicide and crisis interventions on the railways, or to different countries and settings (particularly in unstaffed public locations). Also, although it was beneficial to draw on the experiences of the full range of people who may intervene to prevent a suicide on the railways, the number of front-line staff and commuter interviewees was too small to draw stronger inferences about the differences and similarities between these groups. The decision to focus specifically on the perspectives of bystanders who had intervened to prevent a death on the railways also limits the conclusions that may be drawn regarding barriers to intervention, and when and why helping behaviours may supersede bystander apathy and fear. These questions were, however, explored as part of a broader survey study (reported separately), and lend themselves to further investigation, ideally also involving systematic analysis of naturalistic data, including CCTV footage and official records of railway suicides, attempts and interventions.

The accounts presented in this paper, and their implications, should therefore also be considered alongside wider perspectives (not least those of individuals whose suicide attempts have been stopped or interrupted on the railways, and those who decided not to intervene when witnessing a potential suicide attempt) and data sources. The impact of the COVID-19 pandemic and associated social distancing restrictions on third-party suicide interventions is a further area for research.

In conclusion, for every suicide on the railways, at least six potential attempts are interrupted by front-line staff and rail commuters.^2^ Understanding the experiences of those involved in such interventions can offer important insights and learning, and inform strategies to increase their likelihood, effectiveness and safety, for all concerned.

Although not all interventions with people in distress on the railways may prove to be ‘life-saving’ as such, our study supports earlier evidence in showing that there is a multitude of ways in which bystanders can help individuals in crisis at public locations, including from afar (e.g. by notifying emergency services) and more directly (verbally or non-verbally). This includes members of the general public with little or no training in suicide prevention, although some training or at least guidance in ‘managing suicidal contacts’ appears to increase preparedness to intervene by enhancing confidence levels.

In the specific context of the UK railway network (which arguably differs from other public locations in important ways), our findings suggest that guiding the public about what to do in emergency situations from a safety perspective (e.g. where emergency buttons are located and how to alert a member of staff) is also important, particularly as there might be very little time to intervene when someone is in danger. An advantage of focusing on general emergency situations (rather than in relation to suicide prevention) is to avoid reinforcing cognitive and cultural associations between railway environments and suicides, or indeed suicide interventions. Suggestions that support is available in these environments may inadvertently heighten expectations of intervention, which may not always be possible, and may signal that suicides by train are common, therefore potentially increasing the cognitive availability of this method. The potential for these and other unintended harmful consequences needs careful consideration in designing and evaluating any intervention to reduce suicide (including by third-party interventions), and in ‘advertising’ (or not) any such measure to the general public. Ideally, this should be done in consultation with people with lived experience, as well as academic experts, public health and industry stakeholders, and front-line staff.

## Data Availability

To protect participants’ anonymity, raw interview data cannot be made available upon request.

## Supplementary material

For supplementary material accompanying this paper visit http://doi.org/10.1192/bjo.2022.27. click here to view supplementary material
