# Supplementary material for: Intervening to prevent suicide at railway locations: findings from a qualitative study with front-line staff and rail commuters
Source: BJPsych Open. 2022 Mar 9;8(2):e62. doi: 10.1192/bjo.2022.27 (PMC8935935; doi:10.1192/bjo.2022.27)
Supplement: Supplementary file 1 [file S2056472422000278sup001.docx]

**Consolidated criteria for reporting qualitative studies (COREQ): 32-item checklist**

Developed from:

Tong A, Sainsbury P, Craig J. Consolidated criteria for reporting qualitative research (COREQ): a 32-item checklist for interviews and focus groups. *International Journal for Quality in Health Care*. 2007. Volume 19, Number 6: pp. 349 – 357

| **No. Item** | **Guide questions/description** | **Reported on Page #** |
| --- | --- | --- |
| **Domain 1: Research team and reﬂexivity** |  |  |
| *Personal Characteristics* |  |  |
| 1. Inter viewer/facilitator | Which author/s conducted the inter view or focus group? | Dafni Katsampa |
| 2. Credentials | What were the researcher’s credentials? E.g. PhD, MD | MSc Clinical Mental Health Sciences |
| 3. Occupation | What was their occupation at the time of the study? | Research Assistant |
| 4. Gender | Was the researcher male or female? | Female |
| 5. Experience and training | What experience or training did the researcher have? | MSc Clinical Mental Health Sciences; extensive clinical and research experience; interview techniques. |
| *Relationship with participants* |  |  |
| 6. Relationship established | Was a relationship established prior to study commencement?  *The interviewer introduced themselves prior to the interview and discussed the research with the participant.* | No |
| 7. Participant knowledge of the interviewer | What did the participants know about the researcher? e.g. personal goals, reasons for doing the research  *Participants were told of the interviewer’s professional background and experience, reasons for conducting the research, and funding source.* | Professional role – research assistant in this study |
| 8. Interviewer characteristics | What characteristics were reported about the inter viewer/facilitator? e.g. Bias, assumptions, reasons and interests in the research topic | See column 2  DK is also a Listening Volunteer at the Listening Place |
| **Domain 2: study design** |  |  |
| *Theoretical framework* |  |  |
| 9. Methodological orientation and Theory | What methodological orientation was stated to underpin the study? e.g. grounded theory, discourse analysis, ethnography, phenomenology, content analysis | See manuscript |
| *Participant selection* |  |  |
| 10. Sampling | How were participants selected? e.g. purposive, convenience, consecutive, snowball | See manuscript |
| 11. Method of approach | How were participants approached? e.g. face-to-face, telephone, mail, email | See manuscript |
| 12. Sample size | How many participants were in the study? | See manuscript |
| 13. Non-participation | How many people refused to participate or dropped out? Reasons?  *Refusal to participate: n/a (given recruitment method). No participant dropped out.* | See column 2 |
| *Setting* |  |  |
| 14. Setting of data collection | Where was the data collected? e.g. home, clinic, workplace | See manuscript |
| 15. Presence of non-participants | Was anyone else present besides the participants and researchers? | No. In all interviews that took place face to face at a Samaritans branch, a Samaritans volunteer was available in case the participant needed support prior, during or after the interview. |
| 16. Description of sample | What are the important characteristics of the sample? e.g. demographic data, date | See manuscript |
| *Data collection* |  |  |
| 17. Interview guide | Were questions, prompts, guides provided by the authors? Was it pilot tested? | See supplementary online materials. Interviews were not formally piloted, but reviewed and refined in consultation with the project’s advisory group (which included people with lived experience of suicidality). |
| 18. Repeat interviews | Were repeat interviews carried out? If yes, how many? | No |
| 19. Audio/visual recording | Did the research use audio or visual recording to collect the data? | All interviews were audio-recorded. |
| 20. Field notes | Were ﬁeld notes made during and/or after the inter view or focus group? | Yes |
| 21. Duration | What was the duration of the inter views or focus group? | 37-112 minutes |
| 22. Data saturation | Was data saturation discussed?  *Yes. Despite small numbers, it was felt that this was reached (in relation to each participant group).* | See column 2 |
| 23. Transcripts returned | Were transcripts returned to participants for comment and/or correction?  *This option was offered to all participants, but only taken up by one interviewee.* | See column 2 |
| **Domain 3: analysis and ﬁndings** |  |  |
| *Data analysis* |  |  |
| 24. Number of data coders | How many data coders coded the data? | See manuscript |
| 25. Description of the coding tree | Did authors provide a description of the coding tree? | See manuscript |
| 26. Derivation of themes | Were themes identiﬁed in advance or derived from the data? | See manuscript |
| 27. Software | What software, if applicable, was used to manage the data? | NVivo10 |
| 28. Participant checking | Did participants provide feedback on the ﬁndings?  *Participants did not provide feedback on the findings, but summaries of key findings were made available to all, and sent out to those who had requested this in advance.* | See column 2 |
| *Reporting* |  |  |
| 29. Quotations presented | Were participant quotations presented to illustrate the themes/ﬁndings? Was each quotation identiﬁed? e.g. participant number | See manuscript |
| 30. Data and ﬁndings consistent | Was there consistency between the data presented and the ﬁndings? | See manuscript |
| 31. Clarity of major themes | Were major themes clearly presented in the ﬁndings? | See manuscript |
| 32. Clarity of minor themes | Is there a description of diverse cases or discussion of minor themes? | See manuscript |
